# Methyl-CpG-binding protein 2 (MECP2) mutation type is associated with bone disease severity in Rett syndrome

**DOI:** 10.1186/s12881-020-0960-2

**Published:** 2020-01-31

**Authors:** Carla Caffarelli, Stefano Gonnelli, Maria Dea Tomai Pitinca, Silvia Camarri, Antonella Al Refaie, Joussef Hayek, Ranuccio Nuti

**Affiliations:** 1 Department of Medicine, Surgery and Neuroscience, University of Siena, Policlinico Le Scotte, Viale Bracci 2, 53100 Siena, Italy; 2 Paediatrics Neuropsychiatry Unit, Azienda Ospedaliera Universitaria Senese, Siena, Italy

**Keywords:** Rett syndrome, Methyl-CpG-binding protein 2 (MECP2), Mutation severity, Osteoporosis, Fractures, Scoliosis

## Abstract

**Background:**

More than 95% of individuals with RTT have mutations in methyl-CpG-binding protein 2 (MECP2), whose protein product modulates gene transcription. The disorder is caused by mutations in a single gene and the disease severity in affected individuals can be quite variable. Specific MECP2 mutations may lead phenotypic variability and different degrees of disease severity. It is known that low bone mass is a frequent and early complication of subjects with Rett syndrome. As a consequence of the low bone mass Rett girls are at an increased risk of fragility fractures. This study aimed to investigate if specific MECP2 mutations may affects the degree of involvement of the bone status in Rett subjects.

**Methods:**

In 232 women with Rett syndrome (mean age 13.8 ± 8.3 yrs) we measured bone mineral density at whole body and at femur (BMD-FN and BMD-TH) by using a DXA machine (Hologic QDR 4500). QUS parameters were assessed at phalanxes by Bone Profiler-IGEA (amplitude dependent speed of sound: AD-SoS and bone transmission time: BTT). Moreover, ambulation capacity (independent or assisted), fracture history and presence of scoliosis were assessed. We divided the subjects with the most common point mutations in two group based on genotype-phenotype severity; in particular, there has been consensus in recognising that the mutations R106T, R168X, R255X, R270X are considered more severe.

**Results:**

As aspect, BMD-WB, BMD-FN and BMD-TH were lower in subjects with Rett syndrome that present the most severe mutations with respect to subjects with Rett syndrome with less severe mutations, but the difference was statistically significant only for BMD-FN and BMD-TH (*p* < 0.05). Also both AD-SoS and BTT values were lower in subjects that present the most severe mutations with respect to less severe mutations but the difference was not statistically significant. Moreover, subjects with Rett syndrome with more severe mutations present a higher prevalence of scoliosis (*p* < 0.05) and of inability to walk (*p* < 0.05).

**Conclusion:**

This study confirms that MECP2 mutation type is a strong predictor of disease severity in subjects with Rett syndrome. In particular, the subjects with more severe mutation present a greater deterioration of bone status, and a higher prevalence of scoliosis and inability to walk.

## Background

Rett syndrome (RTT), first reported by the Austrian physician Andreas Rett, is a severe neurological disorder that affects brain development and function in females in approximately 1 in 10,000 live births. In 90–95% of patients diagnosed with classic RTT, the disease is caused by loss-of-function mutations in the X- linked MECP2 gene, which encodes methyl-CpG binding protein 2 (MECP2) [1, 2]. Despite the hundreds of RTT- causing MECP2 mutations that have been identified, eight ‘hotspot’ mutations (encoding the amino acid substitutions R106W, R133C, T158 M, R168X, R255X, R270X, R294X and R306C) constitute more than 60% of documented cases [3]. RTT causing mutations in MECP2 in female patients lead to developmental regression, including a loss of speech and hand skills, after apparently normal development [4].

It’s has been amply reported that females with Rett’s syndrome frequently have marked decreases in bone mineral density (BMD) [5–17]. Several studies, have reported that bone mineral density and ultrasonographic parameters (QUS) were significantly lower in Rett subjects than in controls, and that these were influenced by the anthropometric parameters, adequate nutritional intake and mobility capacity [13–15, 17]. As a consequence of the low bone mass, girls with Rett syndrome are at an increased risk of fragility fractures and it has been reported that 25–40% of Rett girls have fracture at some time during their lives [12]. Particularly, individuals with Rett syndrome are prone to have fracture at the lower limbs, especially the femur. Moreover, scoliosis is prominent in Rett syndrome; the overall incidence of scoliosis in subjects with Rett syndrome is estimated at around 64% [18, 19].

The onset of scoliosis in women with Rett syndrome was seen as early as age 1 year and increased dramatically over the first decade. By the age of 13 years, approximately 80% of this population with Rett syndrome had measurable scoliosis, but only 70% of those had undergone surgical correction [18].

Several papers have shown that the degree of clinical severity is influenced by the type of MECP2 mutation. Recent evidence have reported genotype-phenotype correlations in girls with Rett syndrome, and there has been consensus in recognizing that R133C, R294X, R306C, T158 M and 30 truncations are less severe and R106T, R168X, R255X, R270X and large deletions are more severe [3, 4, 20]. In particular, there is a higher risk of fracture in those with R168X or R270X mutation, in the presence of epilepsy, and if certain anti-epileptic medications are used. Moreover, fracture is closely linked with mobility levels and capacity to bear weight-those with less mobility and limited weight bearing capacity are more likely to fracture [16].

This study aimed to investigate whether specific MECP2 mutations may affect the degree of skeletal involvement and the bone status in subjects with Rett syndrome.

## Materials and methods

### Study population

This study was a retrospective survey of 232 patients (age range 4–33 years; mean age 13.8 ± 8.3 yrs) affected by Rett’s syndrome, referred to the Department of Paediatric Neuropsychiatry of Siena. This Department has a long history of research on Rett syndrome and many patients from different parts of Italy undergo a routine annual follow-up examination in Siena. The clinical and genetic diagnosis of Rett syndrome was made according to the internationally accepted diagnostic criteria [2, 4]. Patients were included if they had a mutation in the MECP2 gene, regardless of clinical phenotype (classical or Rett variant). Patients with CDKL5 and FOXG1 mutations (genes known to cause Rett variants), male Rett patients, and patients with MECP2 duplications were excluded. Using their MECP2 mutation status, we separated the participants with the eight most common point mutations in two groups: less severe mutations (R133C, R294X, R306C and T158 M) and more severe mutations (R106T, R168X, R255X and R270X) [3, 4, 20–22].

The study was approved by the Ethics Committee for human investigation of our Institution and informed consent was obtained according to the rules of the Ethics Committee. Questionnaires completed by parents provided information on clinical data, level of mobility, use of anticonvulsants or calcium/Vitamin D supplements, history of fracture and dietary calcium intake of the patients with Rett syndrome.

At the time of the evaluation 159 (68.5%) patients were non ambulatory whereas the other 73 subjects were ambulatory (31.5%). Moreover, scoliosis was found in 120 (51.6%) out of the subjects with Rett syndrome and 48 females with Rett syndrome (20.9%) had experienced a fracture episode.

### Densitometric and ultrasonographic measurements

In all subjects we performed areal bone mineral density (BMD) at femoral sub regions (femoral neck: BMD-FN and total hip: BMD-T), by dual energy X-ray absorptiometry (DXA), (Hologic QDR 4500, Bedford-MA, US). Also whole body scans were carried out in all subjects by dual-energy X-ray absorptiometry using standardized scan protocols. Whole body mineral content (BMC-WB), whole bone mineral density (BMD-WB), fat mass (FM), fat percentage and lean mass (LM) were determined by using the same DXA device. All scans were performed by the same operator while the subjects were wearing light indoor clothing and non removable metal objects. The subjects with Rett syndrome with severe involuntary muscle contractions or uncontrollable movements were lightly sedated with midazolam (0.2 mg/kg/dose) before the scan to prevent repetitive involuntary movements which could invalidate the analysis.

Quality control was performed weekly with a whole body phantom. In our Institution, the precision error for bone mineral density and bone mineral content (BMC) measurements is less than 2.5% for the whole body phantom. Moreover, in all subjects QUS parameters were evaluated at phalanxes by using a QUS device (Bone Profiler, IGEA, Italy). The device used is based on the transmission of ultrasound through the distal end of the first phalangeal diaphysis in the proximity of the condyles of the last four fingers of the hand. Bone Profiler measures the amplitude-dependent speed of sound (AD-SoS, m/s) and some parameters derived from the analysis of the graphic trace of the QUS signal [23]. The AD-SoS and BTT values of patients with Rett syndrome and controls were converted to Z-scores using the normative data obtained from a reference paediatric Italian population [24]. In our Institution the precision of AD-SoS and BTT evaluated in children was 0.7 and 0.8% respectively. In addition, the standardized coefficient of variation (sCV) was calculated for each QUS parameter according to the formula: sCV = CV%/range/mean, where range was the difference between the 5th and the 95th percentile of the population. The sCV were 3.7% for AD-SoS, and 2.6% for BTT.

### Biochemical parameters

In subjects with Rett syndrome and controls, blood samples were also collected under fasting conditions to evaluate serum calcium levels (Ca), phosphate (P) and 25-Hydroxyvitamin D (25OHD). Serum 25OHD was determined by a radioimmunometric method (25-Hydroxyvitamin D, DiaSorin, MN, USA). In our Institution the intra- and inter-assay coefficients of variation for 25OHD were 6.8 and 9.2%, respectively.

### Statistical analysis

The variables normally distributed were expressed as mean ± standard deviation (SD) and the significance between the means was tested using Student’s t-test.

The association between different clinical characteristics and the outcomes were presented in the contingency table and statistically assessed using Chi square test.

Separate multiple linear regression models (method: Stepwise) were used to assess independent predictors of BMD-FN, BMD-TF, AD-SoS and BTT, while age, weight, height, scoliosis, movement capacity, history of fractures and mutations severity included as independent variables in the models. For each model the regression coefficients (b-coefficients) and their 95% confidence intervals were described. The absence of multicollinearity of predictor variables was determined assessing the trend of partial correlations and it was confirmed by the statistics Tolerance and VIF (variance inflation factor).

All tests were two-sided, and *p* < 0.05 was considered statistically significant. All statistical tests were performed using SPSS 10.1 statistical software (SPSS 10.1).

## Results

The most common eight MECP2 mutations grouped on genotype-phenotype severity are reported in Table 1. Clinical characteristics, biochemical, densitometric and ultrasonographic parameters of females with Rett syndrome grouped on the basis of the genotype-phenotype severity are reported in Table 2. As expected BMD-WB, BMD-FN and BMD-TH were lower in subjects with Rett syndrome that present the most severe mutations with respect to subjects with less severe mutations, but the difference was statistically significant only for BMD-FN and BMD-TH (*p* < 0.05). Also the values of BMC-WB and BMC-WB/height were significantly lower in subjects with Rett syndrome who presented the most severe mutations with respect to less severe mutations. Fat mass and lean mass were lower in subjects with more severe mutations, but the difference reached a statistical significance only for lean mass (*p* < 0.05). However, the percentage of fat was higher in subjects with more severe mutations with respect to less severe mutations, even though the difference did not reach any statistical significance.

**Table 1:**
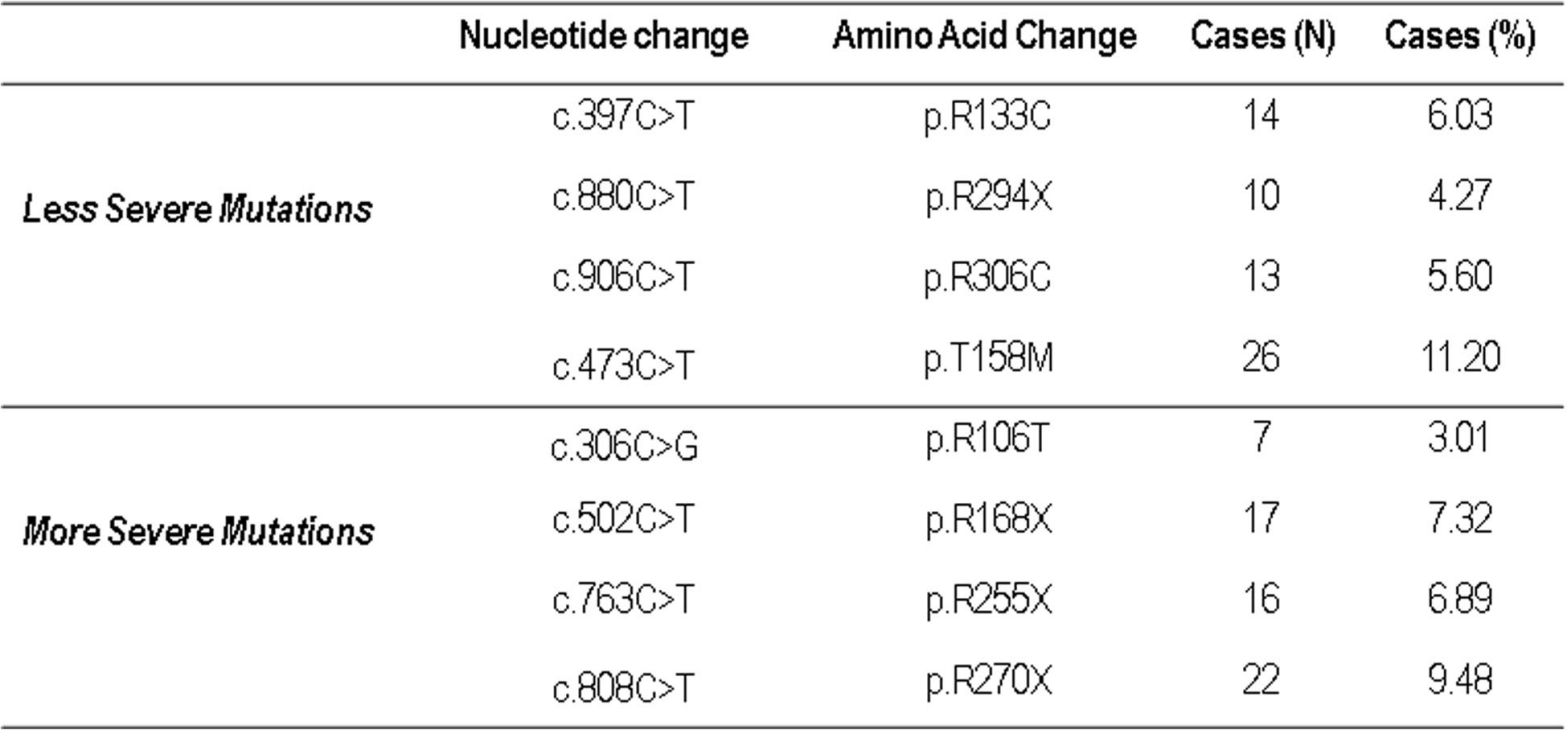
Most common MECP2 mutations in 232 Rett females

**Table 2:**
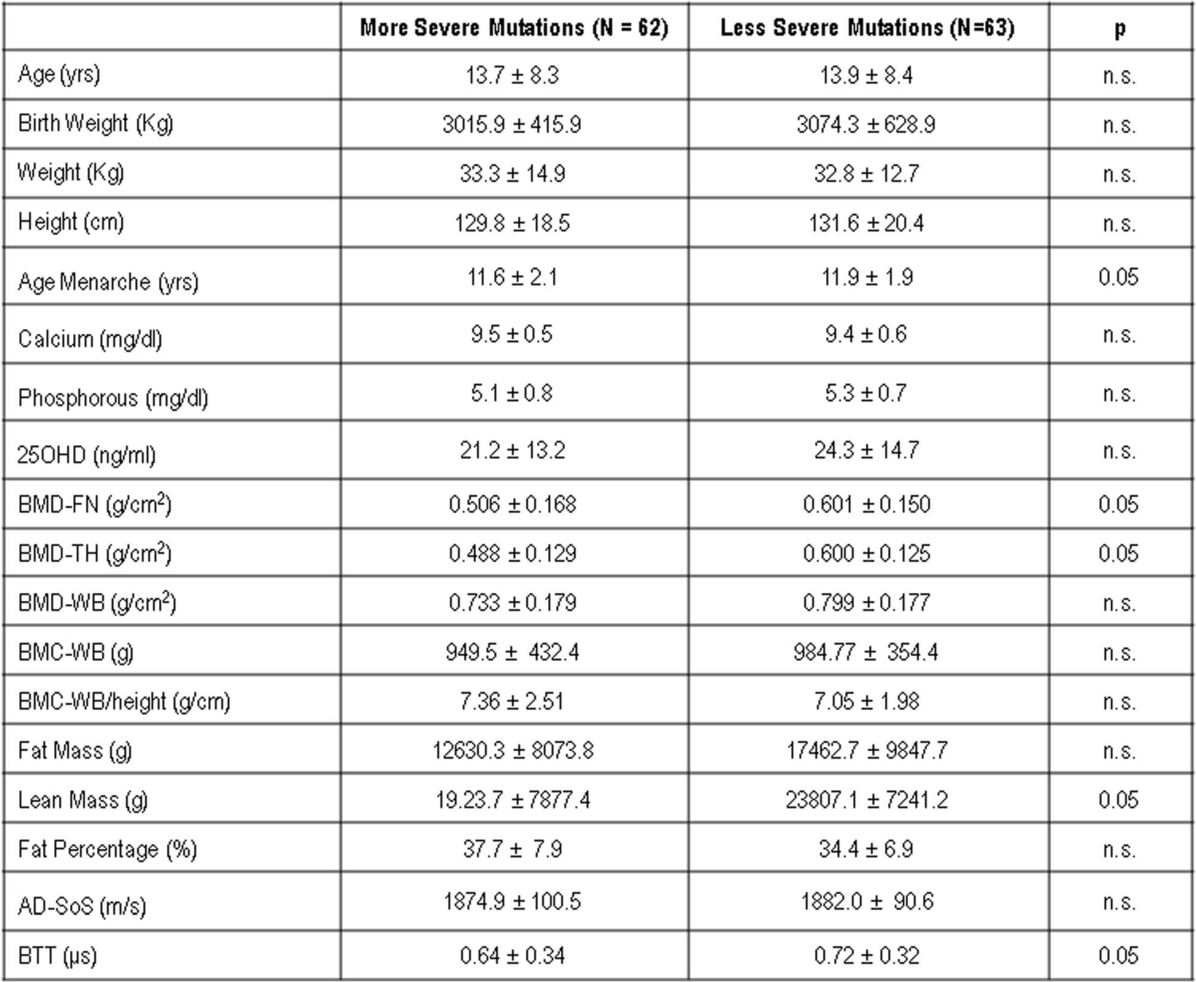
Clinical characteristics in Rett subjects with more or less MCP2 mutations severity

Also, both AD-SoS and BTT values were lower in subjects which presented the most severe mutations with respect to less severe mutations but the difference was statistically significant only for BTT (*p* < 0.05) (Table 2).

**Fig. 1:**
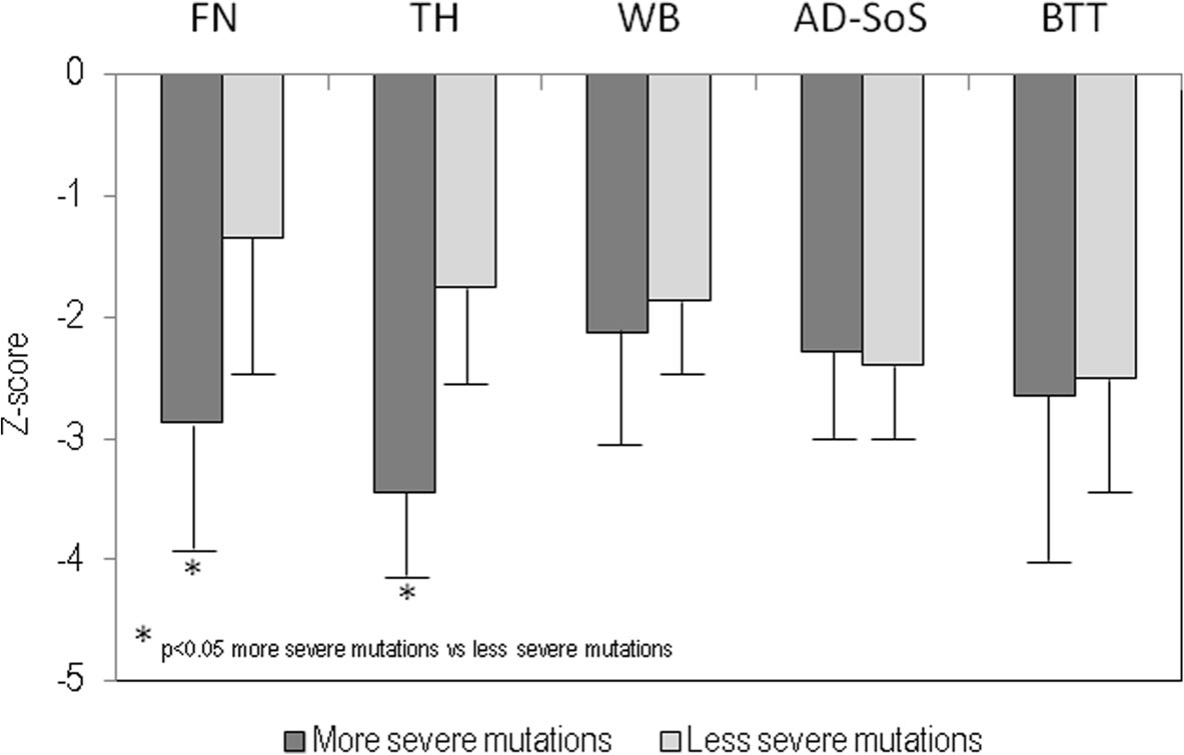
Values of densitometric parameters at femoral neck (FN) and at total hip (TH), whole body (WB) and ultrasonographic parameters at phalanges (AD-SoS and BTT). All values are expressed as Z-score

**Fig. 2:**
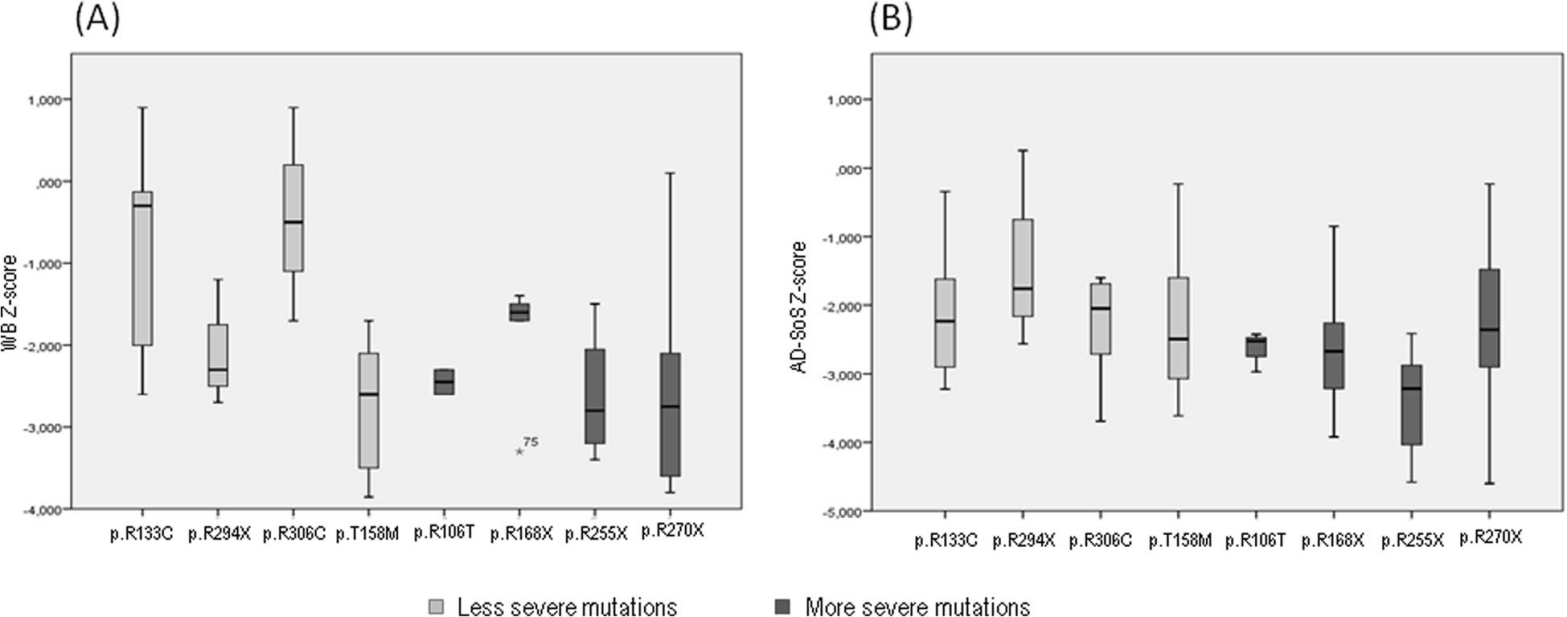
WB Z-score (**a**) and AD-Sos Z-score (**b**) are shown for individual MeCP2 mutations

Figure 1 shows the values of BMD at different skeletal sites and QUS at phalanges, expressed as Z-score in subjects with Rett syndrome grouped on the basis of genotype-phenotype severity; here too, Z-score values were lower in subjects that presented the most severe mutations with respect to those with less severe mutations, but the difference was statistically significant only for Z-score values at total femur (*p* < 0.05). Moreover, WB-BMD Z-score and AD-SoS Z-score values were plotted for specific mutations; the lowest values were observed in patients with more severe mutations respect to less severe mutations (Fig. 2).

**Fig. 3:**
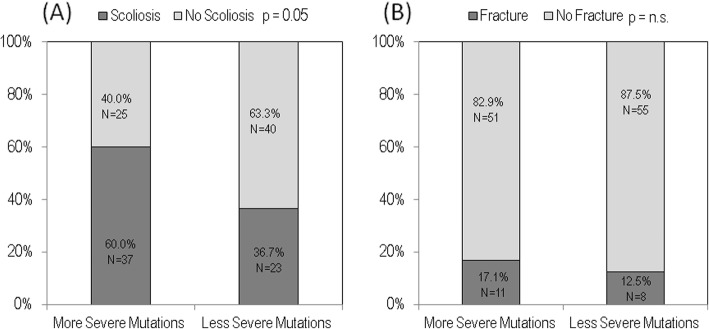
Prevalence of scoliosis (**a**) and fractures (**b**) by mutations severity in the study population

The presence of scoliosis was significantly more frequent in females with more severe mutations respect to less severe mutations subjects (60.0% vs 36.7%, respectively) (Fig. 3a; similarly, the number of girls with Rett syndrome with fractures prevailed in the group with more severe mutations respect to the group with less severe mutations subjects (17.1% vs 12.5%, respectively), but the difference was not statistically significant (Fig. 3b.

**Fig. 4:**
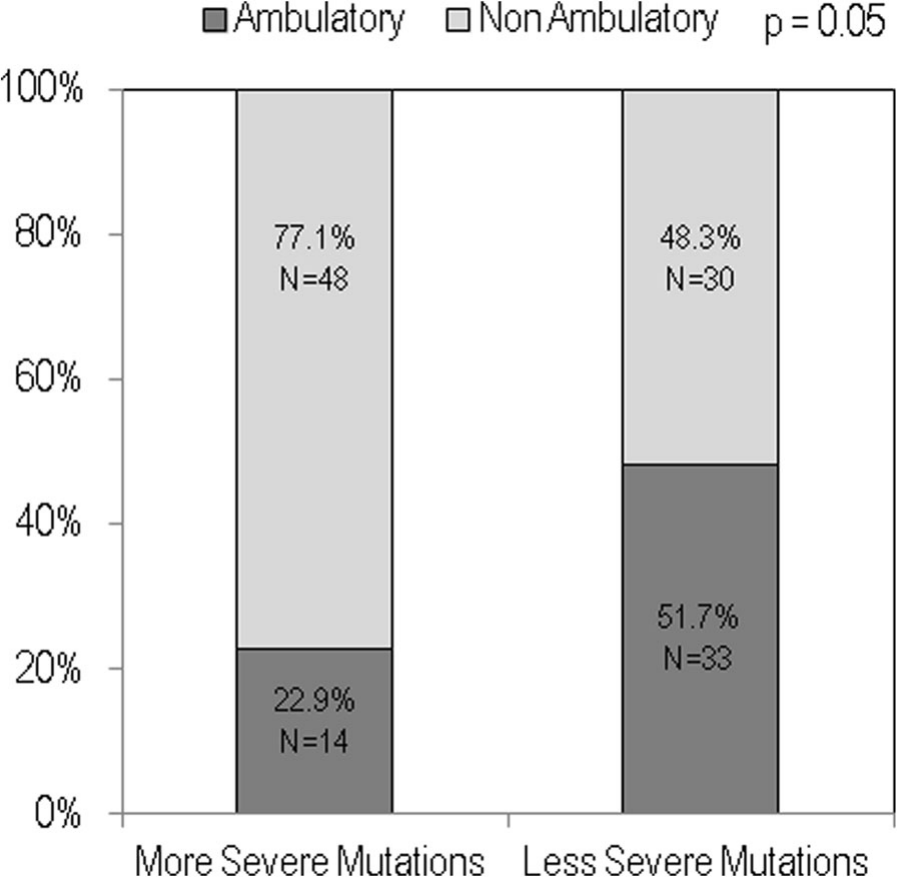
Prevalence of ambulation capacity by mutations severity in the study population

Figure 4 shows that the prevalence of ambulatory patients on the basis of genotype-phenotype severity. The group with more severe mutations showed a significant reduction in females with ambulatory capacity with respect to the group with less severe mutations (22.9% vs 51.7%, respectively).

Multiple linear regression analyses were used to assess independent predictors of bone mineral density and QUS parameters in subjects with Rett syndrome. The analyses was performed by including in the model age, weight, height, movement capacity, scoliosis, history of fracture and mutation severity as independent variables. In Rett subjects BMD-TH was predicted by mutation severity and weight, whereas WB-BMD was predicted by height and history of fracture. Moreover, BTT was predicted by height and age, while AD-SoS was predicted only by age (Table 3).

**Table 3:**
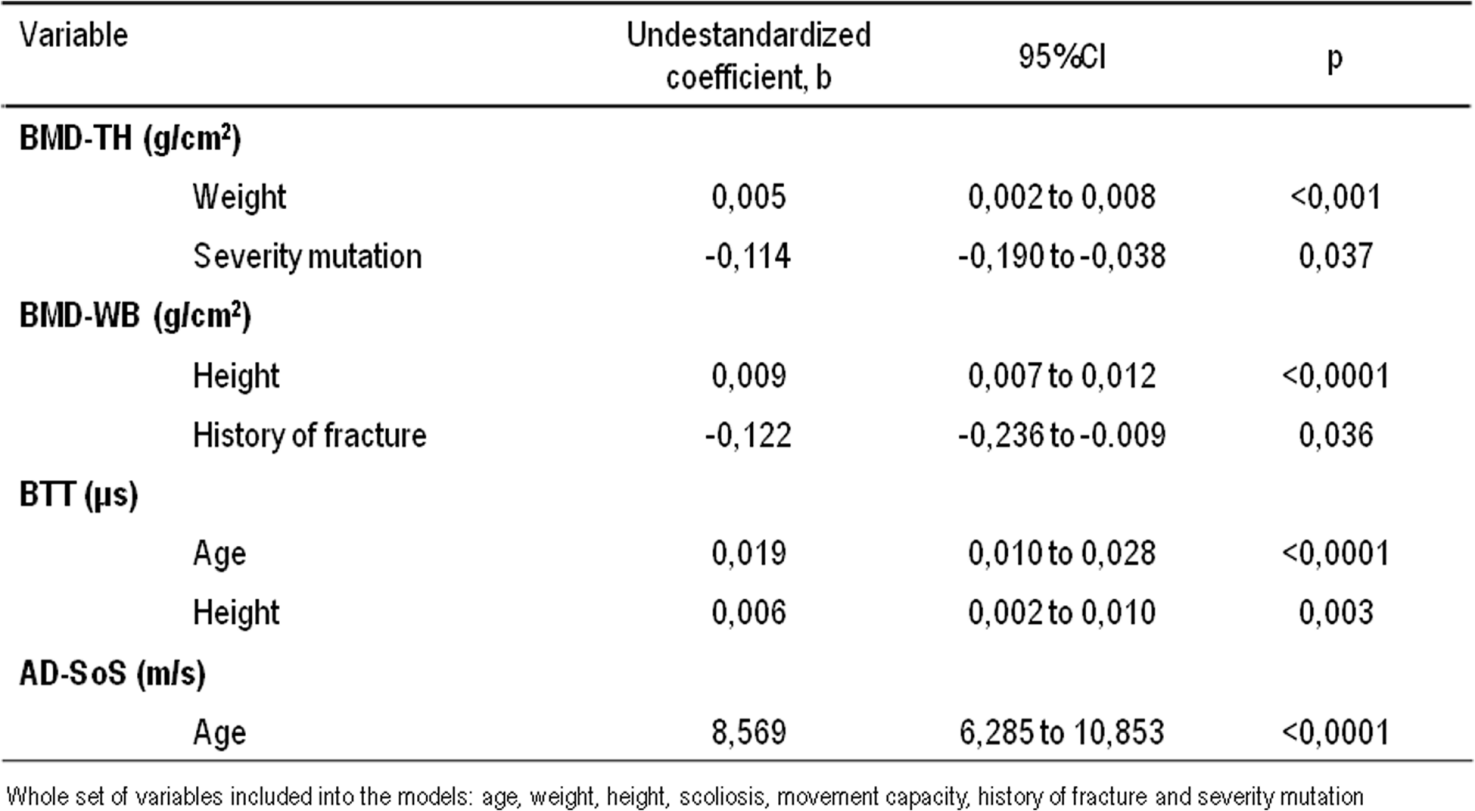
Multiple linear regression analysis of predictors of bone mineral density and QUS parameters in subjects with Rett syndrome

## Discussion

To our knowledge, this study is the first to assess the contribution of specific MECP2 mutations to bone mineral density, presence of fractures, presence of scoliosis and ambulatory capacity in subjects with Rett syndrome.

Several recent reports have identified key associations between MECP2 mutation type and phenotype [3, 4, 20–22, 24–29]. In particular, the majority of these reports have been in agreement in correlating disease severity with particular mutations. For example, R133C, R294X, R306C and large deletions and 30 truncations [3, 20, 21, 26] have all been identified as less severe mutations with lower clinical severities as measured by a variety of clinical severity scales. Moreover, the mutations R106T, R168X, R255X, R270X, splice sites, large deletions, insertions and deletions as being mutations associated with a more severe disease course in subjects with Rett syndrome have been implicated as more severe mutations [3, 20, 21]. The data of the present study are in agreement with all of these findings.

Osteopenia and fracture occur commonly in subjects with Rett syndrome [5–17]. At first, the bone involvement in subjects with Rett syndrome has been explained by more common skeletal risk factors including nutritional deficits, reduced mobility, increased levels of inflammatory cytokines and exposure to osteotoxic drugs such as anti-epileptic treatments [9, 11, 13].

More recent studies have been conducted in order to explore whether MECP2 protein deficiency results in altered structural and functional properties of bone and to test the potential reversibility of any defects. In particular, a series of histological, imaging and biomechanical tests of bone have been carried out in a functional knockout mouse model of RTT [30, 31]. These studies have identified a range of anatomical, biomaterial and biomechanical abnormalities in bone of MECP2-deficient mice and have shown that many of these features are potentially reversible by reactivating the MECP2 gene, even in fully adult mice [30, 31]. These results suggest that bone phenotypes may be important yet tractable features of Rett syndrome [30]. Another recent study carried out in a murine model has reported that osteoblast morphology was altered in the presence of MECP2 deficiency [32].

Our data show that Rett subjects with more severe MECP2 mutation present a greater deterioration of bone status. These findings are in agreement with the previous study by Shapiro et al. which assessed the correlations of bone mass with respect to clinical parameters and mutations involving the MECP2 gene. This report showed that the lowest BMD values were observed in Rett patients with either the T158 M or R270X mutations [9]. Moreover, a relationship of BMD to the T158 M mutation may lie in the observation that individuals with T158 M mutations are more neurodevelopmentally affected than those with other mutations [22]. Also in a case report Hofstaetter JG et al. [32] investigated for the first time bone matrix mineralization in a patient with RTT with a severe typical MECP2 mutation (c.502 CNT, p.R168X) and found that this patient show very low values of BMD and presence of severe scoliosis. According to our data also the study by Jefferson AL et al. found that individuals with the p.R168 and p.R270 mutations presented the greatest clinical severity, including lower mobility and a high prevalence of epilepsy but most of all, presented the most severe bone mineral phenotype [10].

Our data show that the number of subjects with fractures prevailed in females with Rett syndrome with more severe mutations with respect to less severe mutations. In a previous study Downs et al. found that the fracture rate in this population with Rett syndrome was four times that of the general population and that those with the p.R168 and p.R270 mutations were at particularly increased fracture risk at lower limbs and especially at femur [8].

A study by Sarajlija et al. which explore the correlation between demographic and clinical characteristics of patients with Rett syndrome and vitamin D levels showed that females with Rett syndrome with a R255X mutation had an 8.67 times higher probability of experiencing a bone fracture than patients with other mutations [33]. On the contrary the study by Roende G et al. found no association between the mutation types and fracture distribution [12].

Our data show that the presence of scoliosis was significantly more frequent in females with more severe mutations respect to subject with less severe mutations. These findings are in accordance with the study by Killian JT et al. which assessed the onset and progression of severe scoliosis in 913 Rett subjects. In this study, severe scoliosis was found in 251 participants and severe MECP2 mutations (R106W, R168X, R255X, R270X, and large deletions) prevailed in Rett subjects with scoliosis [18]. In addition, Ager et al. in a previous study showed that subjects with R294X and R306C in the MECP2 gene are related to mild developmental problems and to a decreased risk of scoliosis development [34].

Determining associations between MECP2 mutation type and clinical severity is important for several reasons: firstly, genotype-phenotype associations may reveal important molecular insight into MECP2 protein function; secondly, understanding the relationships between mutation types and bone disease severity may enable healthcare providers to better counsel individuals regarding bone disease prognosis; finally, determining the average severity and variance among mutations will allow researchers conducting clinical trials to adjust their inclusion criteria and outcomes based on relative severity.

## Conclusions

In conclusion this study confirms that MECP2 mutation type is associated with clinical severity and influences many aspects of the phenotype including a greater deterioration of bone status, namely BMD at femur, and a higher prevalence of scoliosis and inability to walk. The different functionality of MECP2 suggests there are many downstream pathways that are interesting for understanding the pathophysiology of Rett syndrome, and allowing a search for improve clinical management.

## Data Availability

The datasets used and/or analyzed during the current study are available from the corresponding author on reasonable request.

## Abbreviations

25OHD: 25-Hydroxyvitamin D
AD-SoS: Amplitude dependent speed of sound
BMC: Bone mineral content
BMD: Bone mineral density
BMD-FN: Bone mineral density at femoral neck
BMD-TH: Bone mineral density at total hip
BMD-WB: Bone mineral density at whole body
BTT: Bone transmission time
Ca: Calcium levels
CDKL5: Cyclin-dependent kinase-like 5
DXA: Dual X-ray Absorptiometry
FM: Fat mass
FOXG1: Forkhead box protein G1
LM: Lean mass
MECP2: Methyl-CpG-binding protein 2
P: Phosphate
QUS: Ultrasonographic parameters
RTT: Rett syndrome
sCV: Standardized coefficient of variation
SD: Standard deviation
